# Regulation of Metabolic Disease-Associated Inflammation by Nutrient Sensors

**DOI:** 10.1155/2018/8261432

**Published:** 2018-07-04

**Authors:** Alex S. Yamashita, Thiago Belchior, Fábio S. Lira, Nicolette C. Bishop, Barbara Wessner, José C. Rosa, William T. Festuccia

**Affiliations:** ^1^Department of Physiology and Biophysics, Institute of Biomedical Sciences, University of Sao Paulo, 05508000 Sao Paulo-SP, Brazil; ^2^Exercise and Immunometabolism Research Group, Department of Physical Education, Universidade Estadual Paulista (UNESP), 19060-900 Presidente Prudente-SP, Brazil; ^3^School of Sports, Exercise and Health Science, Loughborough University, Loughborough, UK; ^4^Centre for Sport Science and University Sports, Vienna, Austria; ^5^Department of Cell Biology and Development, Institute of Biomedical Sciences, University of Sao Paulo, 05508000 Sao Paulo-SP, Brazil

## Abstract

Visceral obesity is frequently associated with the development of type 2 diabetes (T2D), a highly prevalent chronic disease that features insulin resistance and pancreatic *β*-cell dysfunction as important hallmarks. Recent evidence indicates that the chronic, low-grade inflammation commonly associated with visceral obesity plays a major role connecting the excessive visceral fat deposition with the development of insulin resistance and pancreatic *β*-cell dysfunction. Herein, we review the mechanisms by which nutrients modulate obesity-associated inflammation.

## 1. Introduction

Visceral obesity, the excessive accumulation of fat in the adipose depots located inside the peritoneal cavity, is a major risk factor for the development of several highly prevalent, chronic diseases, namely, type 2 diabetes (T2D), cardiovascular diseases, and some types of cancer, among others [1, 2]. The increased prevalence of visceral obesity in the last decades has dramatically raised the incidence of its associated diseases. It was estimated, for example, that approximately 415 million people aged 20–79 years in the world had diabetes, such numbers that are expected to grow to 642 million in 2040 [3]. Importantly, a large portion of either overweight or obese individuals also present with T2D, supporting the strong association between both diseases [4].

T2D is a heterogeneous disease that features insulin resistance and pancreatic *β*-cell dysfunction as major hallmarks. Insulin resistance is defined as the inability of insulin to properly stimulate glucose uptake in skeletal muscle and adipose tissue and to inhibit hepatic glucose production. Despite of the intense research in this area, there are still some doubts about the exact sequence of events that leads to the development of obesity-associated insulin resistance as elegantly reviewed in [5]. The most traditional hypothesis suggests that either obesity or the intake of obesogenic, hypercaloric diet promotes first insulin resistance, which then results in hyperglycemia followed by hyperinsulinemia. In accordance to this hypothesis, hyperglycemia and a compensatory hyperinsulinemia are major metabolic phenotypes found in the early stages of T2D. Chronically, hyperglycemia may promote, in a more advanced stage of T2D, pancreatic islet damage and a subsequent decline in insulin secretion. Interestingly, recent studies have suggested, based on findings obtained in obese humans and rodents and patients after bariatric surgery, that hyperinsulinemia, instead of hyperglycemia, is the primary event involved in the development of insulin resistance and T2D. In accordance with this hypothesis, either obesity or the intake of an obesogenic, hypercaloric diet enhances *β*-cell insulin secretion and/or reduces its degradation, promoting hyperinsulinemia, which chronically results in insulin resistance and therefore hyperglycemia. Taking into account the strong evidence supporting both of the aforementioned hypotheses, one may argue that the exact sequence of events involved in the development of obesity-associated insulin resistance and T2D may vary according to the underlying obesogenic conditions.

T2D results from a complex interaction between genetic and environmental factors. Studies evaluating familial risks indicate that T2D has a high 50% heritability, in which individuals with either one affected first-degree relative or at least two affected siblings, independently of the parental diabetes status, are at the higher risk of developing T2D [1]. Genome-wide association studies have identified so far more than 40 diabetes-associated loci, which are related to *β*-cell function, insulin sensitivity, and obesity and respond to approximately 10% of T2D heritability [1]. Noteworthily, the greatest majority of T2D cases are associated with two or more genetic mutations (polygenic nature), such alterations that in most cases increase the propensity, but are not sufficient to induce disease development without the contribution of environmental and/or behavioral factors. The very same rationale applies to the development of visceral obesity, a major risk factor for T2D [2].

Inflammation is at the very centre of metabolic diseases such as obesity, T2D, and metabolic syndrome, playing an important role not only in their development but also as a linking factor between them. Indeed, visceral obesity is associated with a chronic inflammatory process of low intensity, defined as metabolic inflammation or “metainflammation” that affects important metabolic tissues such as adipose tissue, liver, skeletal muscle, pancreas, intestines, and hypothalamus, among others [6]. The adipose tissue, for example, the organ that defines obesity, develops upon this condition an inflammatory process characterized by the recruitment, infiltration, and polarization of leukocytes to a proinflammatory profile [6]. Among the leukocytes recruited to adipose tissue upon obesity are neutrophils, macrophages, dendritic and mast cells from the innate immune system, and several subtypes of T and B lymphocytes from the adaptive immune system [7]. Several stimuli were suggested to mediate the induction of leukocyte recruitment to adipose tissue found upon obesity such as the following: (1) activation of adipocyte proinflammatory pathways by LPS and saturated fatty acids and secretion of chemokines such as monocyte chemoattractant protein-1 (MCP-1), (2) tissue hypoxia, (3) adipocyte death, and (4) mechanical stress between adipocytes and the extracellular matrix [6]. Importantly, after recruitment, tissue leukocytes also undergo polarization to different phenotypes. Macrophages, for example, which account for approximately 50% of the cells composing adipose tissue upon obesity, undergo during this condition a polarization to a proinflammatory M1 phenotype [8]. These proinflammatory leukocytes along with activated, hypertrophied adipocytes secrete a plethora of proinflammatory cytokines/adipokines, chemokines, and lipids, perpetuating inflammation and impairing tissue metabolism. Importantly, proinflammatory mediators have been shown to induce insulin resistance by impairing several steps in the intracellular signaling cascade of this hormone [9, 10] and adipose tissue macrophage depletion attenuates diet-induced obesity, inflammation, and insulin resistance [8].

Intake of excessive amounts of nutrients is a major underlying cause of obesity and obesity-associated complications and an important modulator of many phenotypes associated with this condition including inflammation. We review herein the mechanisms by which excessive intake of nutrients chronically modulates visceral obesity-associated inflammation with a special emphasis in the role of nutrient sensors as likely mediators of these actions.

### 1.1. Nutrient Sensing

Nutrients have vital functions in cells acting not only as metabolic substrates for energy production but also as building blocks for the synthesis of macromolecules and cellular components [11]. In the face of such essential roles of nutrients, organisms have evolved several mechanisms to sense levels of specific nutrients in the extra- and intracellular compartments allowing the proper coordination of rates of growth, proliferation, and function according to nutrient availability [11]. Nutrient-sensing mechanisms are found in all organisms; some of them are well conserved through evolution being found in eukaryotic organisms varying from yeast to mammals, whereas others are exclusive of prokaryotes [11]. Importantly, in multicellular organisms, some nutrient sensing mechanisms also evolved to undergo regulation by the endocrine system, allowing the coordination at whole-body level of nutrient sensing activity among different cells/tissues, which is of major importance in the maintenance of whole-body homeostasis.

Proteins that have the capacity to sense fluctuations in the concentration of a specific nutrient or a product of its metabolism within the physiological range are denominated as nutrient sensors [11]. Nutrient sensors, which can be located at different cell compartments as the plasma membrane, cytosol, organelle endomembranes, or the nucleus, respond to fluctuations in nutrient levels through diverse mechanisms varying from the activation of phosphorylation cascades, changes in gene transcription, and enzymatic activities, among others. Upon obesity, nutrient sensors are chronically challenged by excessive amounts of some nutrients, which impact their activity and regulatory role over cellular processes. More specifically, metabolomics studies have found that visceral obesity is frequently associated with increased serum concentrations of glucose, branched-chain and aromatic amino acids (BCAA and AA, resp.), and several lipids such as saturated and omega 6 fatty acids, acylcarnitines, and phospholipids, among others [12]. Chronic tissue exposure to excessive amounts of some of these nutrients has been implicated in the development of common features of obesity and T2D such as organelle dysfunction (endoplasmic reticulum and mitochondria stress) and oxidative stress, both of which being candidates as triggering factors of obesity-associated inflammation and insulin resistance. On the other hand, elevated intake of some nutrients such as *ω*-3 polyunsaturated fatty acids was shown to protect from obesity-associated inflammation and insulin resistance.

### 1.2. Insulin Signaling and Inflammation

Insulin is a major anabolic hormone that exerts its actions by interacting with a receptor constituted of two extracellular *α*-chains containing the ligand-binding site and two transmembrane *β*-chains that possess the tyrosine kinase activity. The insulin receptor (IR) displays 50% homology to the insulin-like growth factor receptor (IGFR), and in some cells, both receptors may form IR-IGFR hybrids that recognize both insulin and IGF-1 as ligands [13, 14]. Upon interaction with insulin or IGF-1, the receptor changes its conformation and phosphorylates at tyrosine residues itself and a family of adaptor proteins known as insulin receptor substrates (IRS). Tyrosine-phosphorylated IRS then binds to the SH2 domain of the p85 regulatory subunit of the lipid kinase phosphoinositide-3-kinase (PI3K) promoting the conversion of the membrane lipid phosphatidylinositol 3,4-biphosphate (PIP2) to phosphatidylinositol 3,4,5-biphosphate (PIP3). PIP3 then binds to and recruits protein kinase B/Akt to the cell membrane promoting its phosphorylation at Ser473 and Thr308 by the mechanistic target of rapamycin complex 2 (mTORC2) and phosphoinositide dependent-kinase 1 (PDK1), respectively [15]. Upon its phosphorylation and activation, Akt promotes glucose uptake by phosphorylating Rab GAP TBC1D4 (AS160) inducing therefore the translocation of vesicle-containing glucose transporter 4 (GLUT4) to the plasma membrane [16] and inhibits hepatic gluconeogenesis by phosphorylating and inactivating the transcription factor forkhead box protein O1 (FoxO1) and the CREB-CBP-CRTC2 complex, thus suppressing the expression of gluconeogenic enzymes phosphoenolpyruvate carboxykinase (PEPCK) and glucose 6-phosphatase (G6Pase) [15, 17]. In addition to those actions that are susceptible to developing resistance upon obesity and T2D, insulin, through the sequential PI3K-Akt activation, inhibitory phosphorylation of the complex tuberous sclerosis complex 1/2 (TSC1/TSC2), and activation of Ras homolog enriched in the brain (Rheb), activates the mechanistic target of rapamycin complex 1 (mTORC1), which in turn promotes protein synthesis by phosphorylating the ribosomal protein S6K and eukaryotic translational initiation factor 4E-binding protein 1 (4E-BP), among many other processes [18]. Another important signaling pathway induced by insulin is the mitogen-activated protein kinase (MAPK), reviewed in detail elsewhere [19], which promotes cell proliferation, among other actions.

In healthy cells, intracellular insulin signaling is constantly monitored by several regulatory checkpoints that, through a complex system of negative feedback loops, keeps the level of activity of this signaling pathway within the optimal physiological range. One of these regulatory loops is exerted by mTORC1 downstream substrate S6K1 that, when phosphorylated and activated, catalyzes a negative feedback characterized by the inhibitory phosphorylation of IRS at serine residues, blocking its ability to interact and allosterically activate PI3K [20]. Another feedback loop modulating insulin signaling catalyzed by mTORC1 involves the phosphorylation and stabilization of the adaptor protein GRB10, which binds via SH2 domain to the phosphorylated tyrosine at the insulin receptor blocking the interaction with IRS [21, 22]. In addition, GRB10 may also reduce insulin signaling by promoting IRS ubiquitination via the ubiquitin ligase E2 NEDD4.2 and proteasomal degradation [23]. Importantly, dysfunction of these regulatory systems and feedback loops promotes chronic overactivation of intracellular insulin signaling and is associated with cancer incidence, whereas the opposite, that is, impaired insulin signaling (resistance), defines T2D.

The mechanisms by which cells become refractory to insulin action in obesity are not completely understood. Compelling evidence published in the early 1990s had shed light on this issue not only by establishing obesity as an inflammatory disease but also by indicating that inflammatory mediators may play a key role as linking factors between excessive fat deposition and the development of insulin resistance and other associated diseases [24]. More specifically, it was shown in these studies that the proinflammatory cytokine tumor necrosis factor- (TNF-) *α* is oversecreted by adipose tissue in obesity, promotes insulin resistance in rodents, and correlates with insulin resistance in obese children and adults [24–27]. Furthermore, it was later shown that deletion of TNF-*α* or its receptor protects mice against diet-induced obesity and insulin resistance [28], whereas TNF-*α* blockade by treatment with its monoclonal antibody infliximab improved glucose homeostasis in patients with rheumatic disease [29–32]. In spite of those promising findings, TNF-*α* blockade was not effective in treating insulin resistance in obese patients [33], such findings that were somehow expected considering that inflammation is a complex process characterized by the involvement of many protein and lipid mediators. Indeed, subsequent studies have shown that other proinflammatory mediators such as interleukin- (IL-) 1*β*, IL-6, interferon- (IFN-) *γ*, ceramides, prostaglandins, and lipopolysaccharide (LPS) from the membrane of gram-negative bacteria residing in gut microbiota, among others, are also involved in the development of obesity-associated insulin resistance [34–36]. Among those, obesity-associated elevation in the circulating levels of LPS, defined as metabolic endotoxemia, has been considered as a major triggering factor that links excessive intake of fat and the development of obesity-associated inflammation [35, 37]. Another important inflammatory mediator involved in obesity is IL-1*β*, a cytokine whose secretion requires its processing by caspase-1 as the result of the activation of the nucleotide-binding domain, leucine-rich-containing family, pyrin domain-containing 3 (NLRP3) inflammasome, a multimeric cytosolic protein complex activated by pathogen-associated molecular pattern (PAMP) and danger-associated molecular pattern (DAMP) molecules [38]. Indeed, blockade of IL-1*β* actions by treatment with a receptor antagonist (anakinra) or monoclonal antibody reduces systemic inflammation and improves glycemia and *β*-cell secretory function in T2D patients [34].

One common signaling event that is at the very centre of obesity-associated inflammation is the activation of nuclear factor *κ*-light-chain-enhancer of activated B cells (NF*κ*B), a conserved family of transcription factors that regulate inflammatory processes, immune function, development, and growth [39]. Importantly, chronic NF*κ*B activation may be involved in the development of several diseases in addition to obesity such as T2D and cancer, among others. Five NF*κ*B protein members are expressed in mammalian cells, RelA (p65), RelB, c-Rel, NF*κ*B1 (p50/105), and NF*κ*B2 (p52/100) [39]. In the absence of stimulus, dimers of NF*κ*B protein members are bound to the inhibitor *κ*B (I*κ*B), which maintains the NF*κ*B complex in the cytosol, inhibiting therefore its translocation to the nucleus and transcriptional activity. Proinflammatory mediators activate NF*κ*B through a series of events that involve I*κ*B phosphorylation by I*κ*B kinase (IKK), followed by its ubiquitination and proteasomal degradation, which then release the p50/RelA (p65) dimer for translocation to the nucleus, where it can repress or induce gene transcription by binding to 9-10 nucleotide sequences in DNA promoter regions denominated as NF*κ*B response elements [39].

Proinflammatory mediators through the activation of several signaling pathways such as the canonical toll-like receptor- (TLR-) IKK-NF*κ*B, and the kinases c-Jun N-terminal kinases (JNK), Janus kinase (JAK), and mTOR, among others [10], promote insulin resistance by impairing different steps in the intracellular insulin signaling cascade, namely, by either phosphorylating IRS at inhibitory serine residues, or inhibiting Akt dual phosphorylation and activation, or reducing AS160 phosphorylation and GLUT-4 translocation [9, 10, 40]. Importantly, resistance selectively affects only few processes regulated by insulin such as glucose uptake in myocytes and adipocytes and glucose production in hepatocytes. Other insulin actions such as the activation of protein synthesis and de novo lipogenesis do not seem to be impaired in insulin-resistant conditions [40–42]. Below, we review the molecular mechanisms by which some nutrients modulate obesity-associated inflammation.

### 1.3. Glucose Excess (Glucotoxicity) and Inflammation

Chronic tissue exposure to hyperglycemia, due to the elevated intake of diets rich in simple carbohydrates of high glycemic index and/or impaired glucose homeostasis, is associated with the development of tissue inflammation. It has been extensively shown, for example, in rodent adipose tissue, liver, and pancreas, that high sucrose feeding promotes an inflammatory process characterized by enhanced leukocyte recruitment and polarization to a proinflammatory phenotype, as well as exacerbated cytokine production and secretion [43–47]. Similarly to rodents, acute and persistent hyperglycemia in humans is associated with elevated circulating levels of several proinflammatory cytokines and markers of endothelial and oxidative stress [48, 49]. Furthermore, human and rodent pancreatic *β*-cells exposed to high glucose levels displayed NLRP3 inflammasome and caspase-1 activation and enhanced IL-1*β* production [50]. The underlying mechanisms by which exposure to high glucose levels activates the NLRP3 inflammasome and whether this occurs in other cell types than *β*-cells are unknown and deserve to be investigated. Recent findings stating that mitochondrial dissociation and inhibition of the rate-limiting enzyme of glycolysis hexokinase promote NLRP3-inflammasome activation and IL-1*β* in macrophages [51] open the possibility for the involvement of this enzyme as a mediator of glucose actions towards the NLRP3 inflammasome. Indeed, high glucose levels may inhibit hexokinase by inducing cell accumulation of its allosteric inhibitor glucose 6-phosphate and/or the Krebs cycle intermediate citrate, both of which have been shown to promote inflammasome activation in macrophages [51].

**Figure 1:**
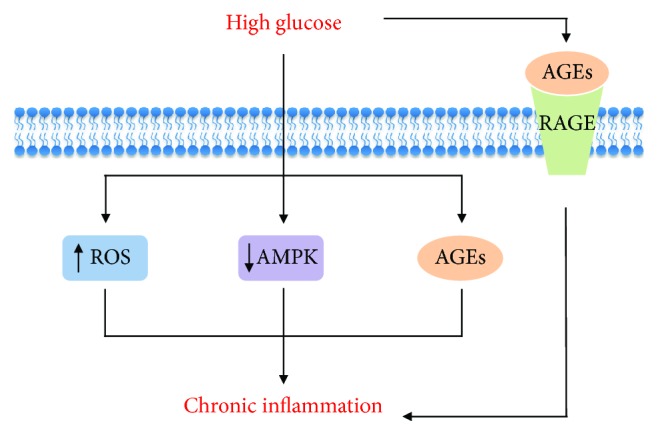
Main mechanisms by which glucose excess induces chronic inflammation. AGEs: advanced glycation end products; AMPK: AMP-activated protein kinase; RAGE: receptor for AGEs; ROS: reactive oxygen species.

In addition to pancreatic *β*-cells, *in vitro* exposure of endothelial cells, monocytes, macrophages, hepatocytes, adipocyte progenitor cells, or mature adipocytes to high glucose levels is associated with a proinflammatory response characterized by activation of the canonical proinflammatory NF*κ*B signaling pathway and elevated cytokine secretion [52–56]. In terms of possible molecular mechanisms, activation of the cellular inflammatory response by high glucose levels has been attributed to the following processes: (1) enhanced production of reactive oxygen species (ROS) and oxidative stress [55], (2) activation of the MAPK pathway [54]. (3) nonenzymatic glycation and formation of advanced glycation end products (AGE) [49], (4) activation of protein kinase C (PKC) isoforms [57], (5) epigenetic and chromatin modifications [52], (6) activation of transforming growth factor- (TGF-) *β*-activated kinase 1 signaling [53], (7) activation of the hexosamine pathway and modification of proteins by N-acetylglucosamine (O-GlcNAc modification) [58], and (8) inhibition of AMP-activated protein kinase (AMPK) [59]. Among those processes, which have been covered before in an excellent review article [60], we will further discuss herein the mechanisms by which exposure to high glucose levels promotes inflammation by inducing oxidative stress and spontaneous glycation and by inhibiting the energy sensor AMPK (Figure 1).

Chronic cell exposure to high glucose levels is associated with an imbalance between ROS production and antioxidant buffering resulting in cellular oxidative stress. Mechanistically, high glucose levels increase ROS production and oxidative stress by enhancing glucose oxidation through the Krebs cycle, NADH/NAD and FADH_2_/FAD ratios and electron flux in a stepwise manner through complexes I and III redox centres, which then catalyzes the transference of some of these electrons to O_2_ [49]. Oxygen radical species are highly reactive substances that when in excess promote organelle dysfunction and cellular damage by reacting with protein, lipids, carbohydrates, and DNA [49]. In oxidative stress, cells trigger an inflammatory response as a protective mechanism to repair cell damage and avoid death that includes activation of intracellular signaling through the NF*κ*B pathway [61], as well as NLRP3 inflammasome-mediated IL-1*β* cleavage and secretion [62, 63].

In addition to oxidative stress, high glucose levels also promote inflammation by enhancing the spontaneous glycation of free amino groups of proteins, DNA, and other molecules, generating, through a series of reactions, AGE [49]. Importantly, glycation is concentration-dependent, does not require enzymatic activity, and, similarly to ROS, impairs the biological function of molecules, inducing organelle dysfunction, stress, and inflammation. In addition to glucose, other simple sugars such as fructose, galactose, and ribose, as well as phosphorylated intermediates of metabolism (glucose 6-phosphate, fructose 6-phosphate, ribose 5-phosphate, etc.), are also precursors for AGE formation [49]. Several circulating proteins such as albumin, insulin, hemoglobin, and the lipoproteins LDL, VLDL, and HDL have also been shown to undergo glycation in conditions of glucose excess, forming precursors of AGE denominated as Amadori products [49]. Among these, glycated albumin and LDL, for example, were shown to induce proinflammatory NF*κ*B signaling, cytokine secretion, and inflammation through the activation of Amadori receptors in the target cells [64, 65]. Similarly to its precursors, AGE exert part of their actions through the activation of a class of ubiquitously expressed membrane receptors of AGE (RAGE). Binding and activation of RAGE by AGE promotes an oxidative stress-dependent proinflammatory cell response characterized by NF*κ*B activation, increased cytokine secretion, activation of cyclooxygenase and prostaglandin synthesis, and enhanced recruitment and activation of both innate and adaptive immune system [49, 66–68]. Supporting this notion, RAGE activation promotes NF*κ*B activation, IL-6 and TNF-*α* secretion, and polarization of bone marrow-derived macrophages to a proinflammatory M1 profile [69]. In contrast, RAGE deletion is associated with reduced leukocyte recruitment and inflammation of the peritoneal cavity in a model of thioglycollate-induced acute peritonitis [70]. Altogether, these findings indicate that modification of cellular constituents by ROS and/or glycation may be responsible, at least in part for the inflammatory response induced by hyperglycemia.

One important nutrient and energy sensor that detects variations in cell glucose availability is the heterotrimeric protein AMP-activated protein kinase (AMPK), a well-conserved, ubiquitously expressed, serine/threonine kinase whose activity is modulated by changes in ADP/ATP ratio and cell energy status. In situations of energy scarcity, AMPK is activated by an elevation in the ADP/ATP ratio, whereas the opposite, that is, a reduction in AMPK activity, occurs in situations of energy surplus and reduced ADP/ATP ratio [71]. Mechanistically, AMPK is activated by ADP and more potently by AMP through a process that involves the binding of adenine nucleotides to the AMPK *γ* subunit. This changes its conformation and leaves it not only prone to the phosphorylation at the *α* subunit Thr172 residue by liver kinase B-1 (LKB-1) but also resistant to the dephosphorylation by protein phosphatase 2A [71]. When active, AMPK promotes glucose uptake, fatty acid oxidation, mitochondrial biogenesis, and other energy-generating catabolic processes and inhibits synthetic pathways, such as fatty acid and protein syntheses [71]. In addition to its role in the regulation of metabolism, several pieces of evidence support a likely involvement of AMPK as an important modulator of obesity- and hyperglycemia-induced inflammation. Under these conditions characterized by energy surplus, AMPK Thr172 phosphorylation and activity are broadly and markedly reduced. Such a phenotype is also seen upon treatment of bone marrow-derived macrophages and dendritic cells with LPS and other proinflammatory molecules [72–75]. Indeed, *in vitro* macrophage AMPK inhibition by either RNAi or expression of AMPK dominant negative or deletion of the AMPK *β*1 subunit is associated with enhanced LPS-induced TNF-*α*, IL-6, and cyclooxygenase-2 levels, diacylglycerol accumulation and PKC activation [72, 74]*. In vivo*, genetic AMPK inhibition in macrophages exacerbates obesity-associated liver and adipose tissue inflammation by increasing macrophage recruitment and polarization to a proinflammatory M1 profile [72]. In accordance with the notion emerged from the above-mentioned loss-of-function studies suggesting that AMPK activity is anti-inflammatory in nature, both *in vitro* and *in vivo* pharmacological activation of this kinase with either AICAR (5-aminoimidazole-4-carboxamide ribonucleotide) or the antidiabetic drugs metformin and troglitazone or the glycolysis inhibitor 2-deoxyglucose is associated with marked attenuation of LPS-induced NF*κ*B activation, as well as iNOS and TNF-*α* expression in myocytes, adipocytes, macrophages, neutrophils, and dendritic cells [75–77]. Furthermore, recent studies have found not only that AMPK activity is increased by the anti-inflammatory molecules IL-10, TGF-*β*, salicylate [74, 78], and adiponectin [79] but also that its constitutive genetic activation in macrophages resulted in impaired production of proinflammatory cytokines and enhanced cell polarization to a M2 anti-inflammatory profile [74]. Mechanistically, AMPK seems to exert its anti-inflammatory actions by impairing intracellular proinflammatory signaling through NF*κ*B, as well as by inducing in macrophages a metabolic shift from aerobic glycolysis to oxidative pathways [74]. Altogether, these findings establish AMPK as an important pharmacological target to counteract obesity and hyperglycemia-associated inflammation.

### 1.4. Amino Acid Excess and Inflammation

Metabolomic characterization of the serum of fasting obese insulin-resistant patients indicates that elevated circulating levels of branched-chain (leucine, isoleucine, and valine) and aromatic (phenylalanine, tyrosine, and tryptophan) amino acids (BCAA and AA, resp.) are not only important metabolic signatures that distinguish obese patients from lean, healthy individuals [12, 80, 81] but also risk factors for the development of insulin resistance and T2D [82, 83]. Despite those findings, it is still unknown whether BCAA have a causal role in the development of obesity-associated insulin resistance or whether elevated circulating levels of BCAA are just a metabolic consequence of this disease. In line with a possible causal contribution, supplementation of a high-fat diet with all three BCAA has deleterious effects on insulin sensitivity in rats [81, 84] and BCAA infusion to healthy man impaired insulin-stimulated glucose disposal in skeletal muscle [85]. Furthermore, treatment of myocytes and murine and human adipocytes with amino acids *in vitro* also induces insulin resistance [85, 86]. Mechanistically, this deleterious effect of BCAA on insulin sensitivity involves the activation of the nutrient and amino acid sensor mTORC1 that promotes insulin resistance by activating S6K1 and therefore the inhibitory phosphorylation of IRS-1 at serine residues. Importantly, mTORC1 is overactivated in adipose tissue, liver, and skeletal muscle of diet- and genetic-induced obese, insulin-resistant rodents [87, 88]. Another mechanism by which mTORC1 overactivation may impair IRS-1 function and induce insulin resistance is through the activation of c-JUN-N terminal kinase (JNK) as the result of exacerbated protein synthesis, endoplasmic reticulum (ER) stress, and unfolded protein response (UPR) [89, 90]. Despite this, it is still unclear whether BCAA are implicated in obesity-associated mTORC1 overactivation and whether this complex is involved in the development of obesity-linked insulin resistance.

In favor of the notion, however, that elevated serum BCAA levels are rather a consequence of obesity and insulin resistance, both inflammation and endoplasmic reticulum stress [91], major hallmarks of these diseases, were shown to increase circulating BCAA levels by reducing rates of BCAA oxidation in adipose tissue and by enhancing skeletal muscle protein degradation [83]. Furthermore, elevated intake of BCAA was associated, independently of genetics, with lower insulin resistance, inflammation, blood pressure, and adiposity-related metabolites in female twins [92]. Such beneficial BCAA actions to metabolic health were also seen in several studies in rodents, where high BCAA intake was associated with reductions in body weight, adiposity, and glucose intolerance in diet-induced obese mice [93, 94]. Along with its beneficial effects on glucose homeostasis, BCAA were shown to exert anti-inflammatory actions. Indeed, elevated intake of BCAA was associated with reductions in muscle damage and inflammation during intensive exercise training [94], in hepatic steatosis and inflammation induced by diet-induced obesity [95, 96], and in chronic white adipose tissue and liver inflammation and early-phase hepatic tumorigenesis associated with obesity [97]. Extending those findings, elevating blood BCAA levels through the deletion of the mitochondrial branched-chain aminotransferase (BCATm) in mice was shown to attenuate the catabolic and proinflammatory effects of LPS and improve survival in response to bacterial infection [98].

Although the mechanisms by which BCAA modulate inflammation are not completely defined, it may involve the activation of the amino acid sensor mTORC1, a multiprotein complex composed by the highly conserved serine-threonine kinase mTOR as its catalytic core, along with the accessory proteins regulatory-associated protein of mTOR (Raptor), mammalian lethal with Sec13 protein 8 (mLST8), DEP domain-containing mTOR-interacting protein (DEPTOR), proline-rich Akt substrate 40 (PRAS40), and Tti1/Tel2 complex proteins [18]. The BCAA leucine activates mTORC1 by inducing its translocation to the lysosomes and interaction with the GTP-bound protein Ras homolog enriched in the brain (Rheb). This requires leucine binding to sestrin 2 and activation of small GTPases denominated as Ras-related GTP-binding protein (Rags), through a process that involves the protein complexes GAP activity towards Rags (GATOR) 1 and 2 and the Ragulator [18, 99]. Importantly, mTORC1's major cellular function is to coordinate processes such as cell growth, proliferation, metabolism, autophagy, and survival according to nutrient and growth factor availability [18].

**Figure 2:**
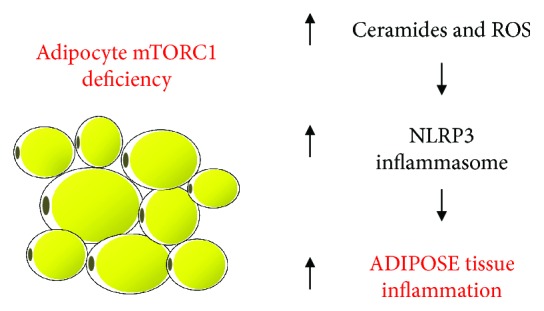
Mechanisms underlying adipose tissue chronic inflammation induced by mTORC1 deficiency in adipocytes. ROS: reactive oxygen species.

In addition to amino acids and growth factors, mTORC1 activity is also upregulated by proinflammatory mediators such as LPS via TLR-4-mediated activation of either IKK*β* [100, 101] or PI3K-Akt [102, 103] and by the anti-inflammatory cytokines IL-4 and IL-13 [104], indicating that this complex may have a role in the regulation of inflammation and immune function. Indeed, pharmacological mTORC1 inhibition with the macrolide rapamycin is associated with an exacerbation of obesity-associated adipose tissue inflammation, as evidenced by the enhanced tissue recruitment and polarization of macrophages to a proinflammatory phenotype and expression of proinflammatory cytokines IL-1*β* and TNF-*α* [104, 105]. In accordance to these findings, mTORC1 inhibition with rapamycin was shown to enhance the spontaneous polarization of human monocytes *in vitro* and peripheral blood mononuclear cells *in vivo* to a proinflammatory profile [106, 107]; such a response is also seen in murine bone marrow-derived macrophages *in vitro* [104]. Importantly, Raptor deletion and therefore mTORC1 deficiency in adipocytes, but not myeloid cells [108], promoted adipose tissue inflammation, NLRP3-inflammasome activation, leukocyte recruitment, and local cytokine production [109]. Interestingly, this inflammatory response, which occurred despite of a drastic reduction in adipose tissue mass, was mediated by an increase in the local production of ceramides [109] (Figure 2).

The effects of mTORC1 constitutive activation in embryonic fibroblasts and macrophages have also been investigated reporting contrasting results. Constitutive mTORC1 activation through the inactivation of either TSC1 or TSC2 in human monocytes, murine myeloid cells, and embryonic fibroblasts was shown to (1) limit the inflammatory response by blocking LPS-induced NF*κ*B activation and increasing IL-10 production [107], (2) induce in a cell autonomous manner granuloma formation and lung and liver infiltration of alternatively activated M2 macrophages and reduce lung iNOS [110], (3) increase the activity of the energy sensor AMPK that exerts anti-inflammatory actions [111], and (4) protect from diet-induced obesity and adipose tissue inflammation by promoting the polarization of adipose tissue-resident macrophages to a M2 phenotype (Paschoal et al., unpublished observations). Noteworthily, a recent study found that the Akt-mTORC1 pathway is an important mediator of macrophage polarization to a M2 phenotype induced by IL-4, through a mechanism that involves activation of ATP-citrate lyase, histone acetylation, and transcriptional induction of a subset of M2 genes [112]. In contrast to those findings, however, *Tsc1*-deficient bone marrow-derived macrophages (BMDM) were refractory to M2 polarization induced by IL-4 and displayed enhanced M1 polarization, nitric oxide (NO) production, and cytokine secretion induced by toll-like receptor ligands [113, 114]. Surprisingly, another study found that *Tsc1*-deficient macrophages exhibited enhanced polarization to both M1 and M2 phenotypes both at steady-state condition and induced by either LPS or IL-4 [115].

**Figure 3:**
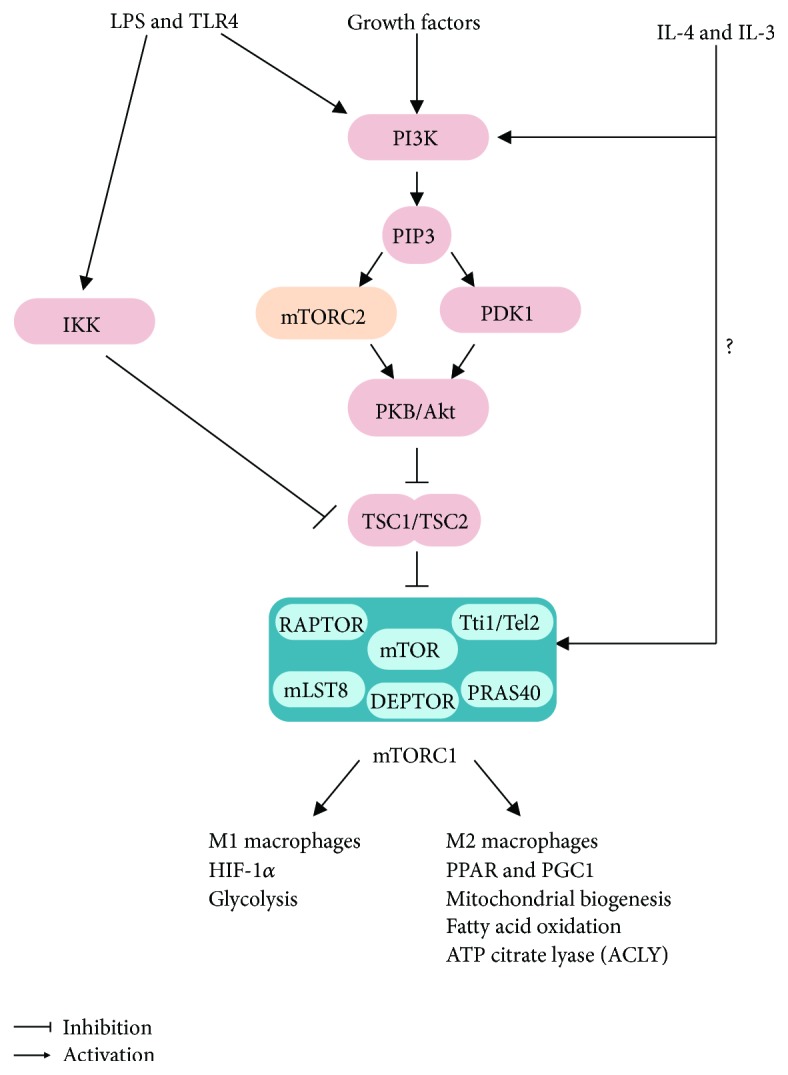
mTORC1 is activated in both M1 and M2 polarization and regulates important metabolic processes at both conditions. AA: amino acids; ACLY: ATP citrate lyase; HIF-1*α*: hypoxic inducible factor 1*α*; IL-4: interleukin 4; IL-13: interleukin 13; PGC1*α*: PPAR*γ* coactivator 1*α*; PPAR*γ*: peroxisome proliferator-activated receptor *γ*; TLR4: toll like receptor 4.

In spite of the apparent contradictory findings, the scenario emerging from the aforementioned studies is that mTORC1 is an important mediator of both proinflammatory M1 and anti-inflammatory M2 macrophage responses (Figure 3). Indeed, this is in accordance with mTORC1's role in the regulation of processes such as protein, lipid and nucleotide syntheses, autophagy, and lysosome formation that are vital to macrophages independently of their phenotype. Accordingly with this notion, mTORC1 activity is increased by proinflammatory molecules (LPS, TNF-*α*, and IFN-*γ*) in M1 macrophage polarization [100, 102, 104], as well as by the M2 macrophage-promoting cytokines (IL-4 and IL-13) [104, 112]. Furthermore, mTORC1 induces metabolic processes that are important for M1 and M2 macrophage functions, namely, aerobic glycolysis and oxidative metabolism, respectively, through mechanisms that involve activation of hypoxic-inducible factor 1*α* (HIF-1*α*) in the former (M1 aerobic glycolysis) and the peroxisome proliferator-activated receptor *γ* (PPAR*γ*), PPAR coativactor 1*α* (PGC1*α*), and mitochondrial biogenesis in the latter (M2 oxidative metabolism) [116]. Altogether, these findings suggest that mTORC1 is an important mediator of both classic M1 and alternative M2 macrophage polarizations, exerting its functions according to the underlying stimuli context (Figure 3). In addition to macrophages, mTORC1 seems to play an important role regulating T lymphocyte proliferation, migration, differentiation, metabolism, and activation as elegantly reviewed before [117].

**Figure 4:**
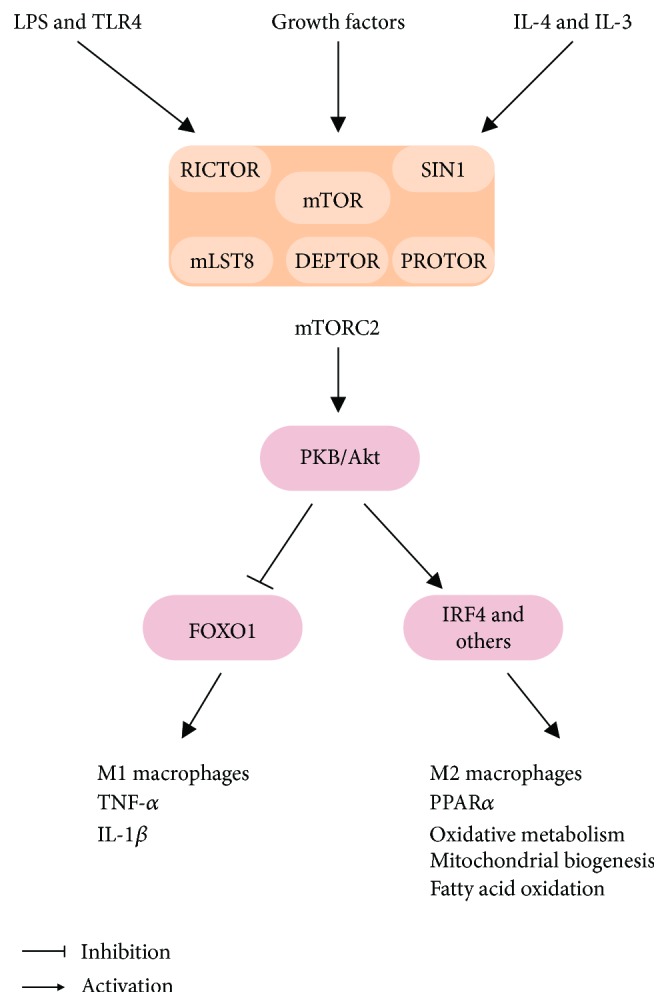
mTORC2 is activated in both M1 and M2 polarization and regulates important metabolic processes at both conditions. IL-1*β*: interleukin 1*β*; IL-4: interleukin 4; IL-13: interleukin 13; IRF4: interferon regulatory factor 4; LPS: lipopolysaccharide; PKB: protein kinase B; PPAR*γ*: peroxisome proliferator-activated receptor *γ*; TLR4: toll-like receptor 4; TNF-*α*: tumor necrosis factor-*α*.

Similarly to complex 1, mTORC2 is also activated by both M1 (LPS and IFN-*γ*) and M2 (IL-4 and IL-13) inducers playing an important role in regulation of macrophage polarization and function (Figure 4). More specifically, deletion of rapamycin-insensitive companion of mTOR (RICTOR) and therefore mTORC2 deficiency in myeloid cells was shown to enhance both polarization of bone marrow-derived macrophages to the M1 profile and the proinflammatory cytokine secretion induced by LPS and other TLR ligands and to reduce the expression of M2-related genes [103]. Furthermore, mice with mTORC2 disruption in myeloid cells had higher circulating TNF-*α* levels and mortality in a model of acute septic shock induced by high dose of LPS [103]. Mechanistically, this enhanced M1 polarization induced by mTORC2 deficiency was attributed to FOXO1 activation due to reduced Akt activity and therefore enhanced transcription of proinflammatory genes [118]. Subsequent studies have also shown that mTORC2 is essential for proper bone marrow-derived macrophage polarization to a M2 profile. Indeed, mTORC2-deficient M2 macrophages were shown to display impaired glucose metabolism and reduced PPAR*γ* content, mitochondrial biogenesis, and fatty acid oxidation, effects that are mediated by Akt and, at least in part, by the transcriptional factor interferon regulatory factor 4 (IRF4) [119].

### 1.5. Fatty Acid Excess and Inflammation

**Figure 5:**
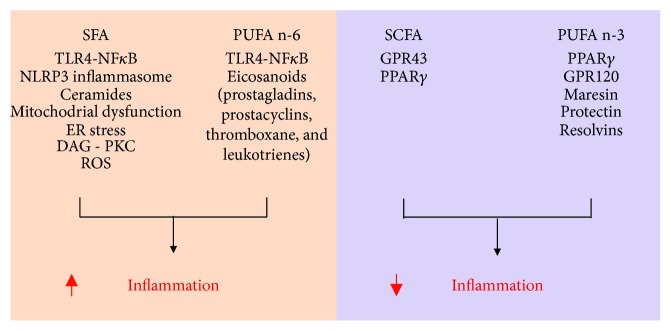
General overview of the mechanisms by which different fatty acids modulate inflammation. AMPK: AMP-activated protein kinase; DAG: diacylglycerol; ER: endoplasmic reticulum; GPR: G protein-coupled receptor; PKC: protein kinase C; PPAR: peroxisome proliferator-activated receptor; PUFA n-6: polyunsaturated n-6 fatty acids; PUFA n-3: polyunsaturated n-3 fatty acids; ROS: reactive oxygen species; SCFA: short-chain fatty acids; SFA: saturated fatty acids; TLR: toll-like receptor.

Evidence suggests that lipids, especially fatty acids, play an important role not only in obesity development but also as a linking factor between the excessive adiposity and development of associated diseases [120]. Indeed, elevated intake of diets containing high amounts of saturated fatty acids induces obesity and its major complications including inflammation, insulin resistance, and ectopic lipid deposition, among others. Recent studies, however, have indicated that more important than quantity, diet fatty acid composition has major implications in the development of “metainflammation.” High intake of diets rich in saturated fatty acids, for instance, activates the innate immune toll-like receptor 4 (TLR4) promoting chronic low-grade inflammation, insulin resistance, and cardiovascular disease [121]. In the same line, *ω*-6 polyunsaturated fatty acid-enriched diets were shown to be proinflammatory and deleterious to the cardiovascular function [121, 122]. In spite of the deleterious effects of saturated and *ω*-6 polyunsaturated fatty acids to metabolic health, other types of lipids have preventive and/or therapeutic properties that could be explored to counteract metabolic diseases. Among beneficial lipids, *ω*-3 polyunsaturated and short-chain fatty acids were demonstrated to have anti-inflammatory, anti-carcinogenic, hypolipidemic, and weight loss-inducing properties [123, 124]. Below, we discuss the modulation of “metainflammation” by specific types of fatty acids (Figure 5).

### 1.6. Saturated Fatty Acids (SFAs)

Saturated fatty acids such as lauric (C12:0), miristic (C14:0), palmitic (C16:0), and stearic (C18:0) are commonly found in foods of animal source such as dairy products and meat, and some vegetables as coconut and palm oil, being therefore the most prevalent saturated fatty acids found in human diet. Many studies have associated an elevated intake of saturated fatty acids with a higher prevalence of obesity, cardiovascular disease, diabetes, and insulin resistance, diseases that share chronic low-intensity inflammation as a common feature [125]. Therefore, since 1961, the American Heart Association recommends a reduction in the intake of foods rich in saturated fatty acids aiming to reduce the prevalence of those chronic metabolic diseases [126].

At the molecular level, saturated fatty acids promote inflammation through several mechanisms that include activation of the TLR4-IKK-NF*κ*B signaling pathway and NLRP3 inflammasome, enhanced ROS production and oxidative stress, mitochondrial dysfunction, ER stress, accumulation of diacylglycerol and ceramides, and PKC activation, among others [63, 127–132]. Saturated fatty acids are potent activators of the pattern recognition receptor TLR4, which is also activated by LPS and others PAMPs and DAMPs and has a major role in the regulation of innate immune response [128, 133, 134]. Activation of TLR4 by saturated fatty acids elicits, through the activation of NF*κ*B, the secretion of several proinflammatory cytokines such as TNF-*α*, IL-6, and MCP-1, which are involved in the development of obesity-associated inflammation and insulin resistance [128, 133, 134]. In addition, saturated fatty acids were shown to activate NLRP3 inflammasome-mediated IL-1*β* production in macrophages, such an effect that seems to involve AMPK inactivation, autophagy inhibition, and enhanced mitochondrial ROS production [129].

In addition to inflammatory signaling, chronic tissue exposure to high levels of saturated fatty acids may promote insulin resistance by increasing tissue content of the lipids diacylglycerol and ceramides. Diacylglycerol accumulation promotes insulin resistance by activating the PKC*Θ* and *ε* impairing IRS tyrosine phosphorylation and therefore downstream insulin signaling [135]. Ceramides, on the other hand, consist of a family of lipids structurally formed by a sphingosine base bound to a fatty acid that promote insulin resistance by two nonmutually exclusive mechanisms, involving either the allosteric activation of protein phosphatase 2A (PP2A), which dephosphorylates and inhibits Akt [136], or the activation of atypical PKC *λ*/*ζ*, which phosphorylates Akt pleckstrin domain at residue 34 impairing its translocation to the membrane and subsequent activation [137, 138]. Ceramides have been also shown to modulate inflammatory processes displaying either pro- or anti-inflammatory responses, as elegantly reviewed before [139]. Briefly, proinflammatory actions of ceramides involve activation of TLR4 signaling as a result of ceramide interaction with this receptor, formation of TLR4-lipid raft complex, and activation of NLRP3 inflammasome-mediated IL-1*β* and IL-18 production [139].

### 1.7. Monounsaturated Fatty Acids (MUFAs)

In contrast to their saturated counterparts, elevated intake of monounsaturated fatty acids is associated with reduced adipocyte hypertrophy, adipose tissue infiltration of proinflammatory macrophages, and inflammation and improved insulin sensitivity [140–142]. In line with these findings, oleic acid (C18:1), the most abundant monounsaturated fatty acid found in human diet, promotes the secretion of the anti-inflammatory adipokine adiponectin and impairs the proinflammatory resistin in 3T3-L1 adipocytes [143]. Furthermore, diet supplementation with macadamia oil, a rich source of oleic acid, improves lipid metabolism and glucose homeostasis in diet-induced obese mice [144, 145]. Although oleic acid is the major fatty acid found in macadamia oil, this nut is also an important natural source of the monounsaturated fatty acid palmitoleic acid (C16:1). This fatty acid, which can be obtained either from diet or from endogenous synthesis by stearoyl-CoA desaturase- (SCD-) 1 mainly in adipose tissue and liver, was shown to enhance whole-body glucose disposal and attenuate hepatic steatosis in diet-induced obese mice [146, 147] and protect *β*-cell from death induced by palmitic acid [148, 149]. In line with those findings, a two-week supplementation with palmitoleic acid improved glucose homeostasis and insulin sensitivity, reduced hepatic steatosis, and increased skeletal muscle fatty acid oxidation in diet-induced obese mice [150]. In addition, palmitoleic acid also increased hepatic oxidative metabolism by activating the AMPK-fibroblast growth factor- (FGF-) 21-peroxisome proliferator-activated receptor (PPAR) *α* axis [151]. In addition to these beneficial metabolic actions, palmitoleic acid was recently shown to have anti-inflammatory properties. Indeed, macrophages pretreated with palmitoleic acid secrete less proinflammatory cytokines after either LPS or palmitic acid treatment [152, 153] and displayed reduced macrophage polarization to a M1 phenotype in part due to AMPK activation [154]. Altogether, these findings support a possible utilization of palmitoleic acid supplementation as a nonpharmacological strategy to reduce obesity-associated chronic low-grade inflammation [155]. Interestingly, palmitoleic acid was also shown to enhance lipolysis, glucose uptake, and GLUT-4 content in adipocytes, such effects that are mechanistically induced by PPAR*α* and AMPK activation, respectively [156, 157]. Whether palmitoleic acid also has anti-inflammatory actions in white adipose tissue needs to be investigated.

### 1.8. *ω*-6 Polyunsaturated Fatty Acids

In addition to the high content of saturated fatty acids, another main feature defining the Western diet is the elevated *ω*-6 and low *ω*-3 polyunsaturated fatty acid contents [158]. Evidence indicates that this high *ω*-6/*ω*-3 ratio dietary pattern is one of the main causes of the rise in obesity and associated metabolic diseases seen in the last decades [159]. Linoleic acid, the most abundant *ω*-6 fatty acid in the Western diet, has important actions on cholesterol metabolism, as well as on inflammation. More specifically, linoleic acid was shown to lower blood cholesterol levels by increasing both hepatic clearance of low-density lipoprotein (LDL) [160] and production of bile acids and to be proinflammatory by activating NF*κ*B in endothelial cells [161], although this does not seem to be reflected by systemic markers of inflammation [162]. Linoleic acid can be enzymatically converted to arachidonic acid, a *ω*-6 fatty acid that acts as the precursor for the synthesis of two classes of lipid mediators known as eicosanoids (prostaglandins, prostacyclins, thromboxane, and leukotrienes) and endocannabinoids (anandamide and 2-arachidonoylglycerol, among others). Eicosanoids are potent regulators of inflammation and immune function, being therefore implicated in the development of chronic metabolic diseases such as obesity, insulin resistance, and cancer [159]. Indeed, recent studies have shown that the eicosanoid leukotriene B4 promotes adipose tissue inflammation by inducing tissue macrophage chemotaxis and polarization to the proinflammatory M1 profile, as well as insulin resistance in the liver and skeletal muscle [163]. Interestingly, opposite effects were seen upon treatment with the eicosanoid lipoxin A4, which reduced adipose tissue inflammation as evidenced by diminished tissue expression of proinflammatory cytokines and higher macrophage polarization to the anti-inflammatory M2 profile [164]. These findings indicate that different eicosanoids have distinct roles in the regulation of adipose tissue inflammation and therefore in the development of obesity-associated insulin resistance.

### 1.9. *ω*-3 Polyunsaturated Fatty Acids

It has been known since the 1980s that fish oil, which is rich in the *ω*-3 polyunsaturated fatty acids eicosapentaenoic acid (EPA) (C20:5, *ω*-3) and docosahexaenoic acid (DHA) (C22:6, *ω*-3), has many beneficial health effects. Indeed, increased whole-body *ω*-3 polyunsaturated fatty acid availability either genetically or through dietary supplementation is associated with hypotriglyceridemia [165], improvement in cardiovascular health by diminishing platelet aggregation and thromboxane levels [166, 167], and attenuation of systemic inflammation [168, 169]. More recently, *ω*-3 polyunsaturated fatty acids were also shown to protect mice from diet-induced obesity, insulin resistance, hepatic steatosis, and tumorigenesis; such effects were attributed at least in part to the anti-inflammatory properties of these fatty acids [123, 170]. Several mechanisms have been proposed to account for the anti-inflammatory actions of *ω*-3 polyunsaturated fatty acids including the activation of the plasma membrane G protein-coupled receptor 120 (GPR120) [171], activation of the nuclear receptor PPAR*γ* [172], inhibition of the conversion of *ω*-6 fatty acids into eicosanoids [159], alterations in plasma membrane composition, fluidity and signaling [173], and induction of the synthesis of proresolution lipid mediators such as resolvins, protectins, and maresins [174]. Among these, recent studies have shown that activation of GPR120, a receptor mainly expressed in adipocytes and macrophages, may be essential to the anti-inflammatory actions of *ω*-3 polyunsaturated fatty acids. Indeed, both EPA and DHA were shown to impair LPS-induced JNK activation and TNF-*α* and IL-6 secretion by RAW 264.7 cells and 3T3-L1 adipocytes *in vitro* through the activation of GPR120 [171]. In line with those findings, mice deficient in GPR120 were resistant to the anti-inflammatory actions and the improvement in whole-body glucose homeostasis induced by the intake of diet rich in *ω*-3 polyunsaturated fatty acids [171]. Two recent studies, however, have challenged the notion that GPR120 activation is mandatory for those *ω*-3 polyunsaturated fatty acid actions. These studies have shown that intake of diet rich in *ω*-3 polyunsaturated fatty acids was equally effective in reducing body weight gain, improving glucose homeostasis and attenuating inflammation in a new generated mice deficient in GPR120 and wild-type controls [175, 176].

Another mechanism by which *ω*-3 polyunsaturated fatty acids may reduce inflammation is by enhancing the synthesis of proresolution lipid mediators, namely, protectins, resolvins, and maresins. Both EPA (e-series) and DHA (d-series) are precursors for the synthesis of these mediators [177], which have been shown to control the magnitude and duration of inflammatory processes in several rodent models of chronic diseases [174]. In line with this notion, the resolvin E1 and protectin D1 were shown to improve insulin sensitivity and reduce hepatic steatosis and adipose tissue inflammation in diet-induced obese mice [178], whereas resolvin D1 improved glucose tolerance, increased adiponectin secretion, and reduced adipose tissue macrophage recruitment and formation of crown-like structures in genetically obese *db/db* mice [179]. Furthermore, protectin DX improved glucose homeostasis in *db/db* mice by promoting IL-6 secretion from skeletal muscle without affecting white adipose tissue inflammation [180]. Further studies characterizing the biological function of already described and new metabolites from EPA and DHA are required to explore the promising usage of these lipids in the prevention and/or treatment of chronic inflammatory diseases.

### 1.10. Short-Chain Fatty Acids (SCFA)

The short-chain fatty acids acetate (C_2_), propionate (C_3_), and butyrate (C_4_) are produced at the intestine by anaerobic fermentation of nondigestible dietary fibers, being readily absorbed and used as energy source by colonocytes and by other body tissues including liver and muscle [181]. In addition to their role as metabolic substrates, short-chain fatty acids regulate several aspects of inflammatory processes such as the recruitment of circulating leukocytes in the inflammatory site, production of chemokines and cytokines, expression of adhesion molecules, production of eicosanoids and reactive oxygen species, and lymphocyte proliferation and differentiation [124]. Studies evaluating the effects of short-chain fatty acids in animal models of inflammatory diseases including acute kidney injury, obesity, and T2D indicate that these molecules are potent anti-inflammatory agents [182, 183]. Indeed, sodium butyrate administration to obese and diabetic *db/db* mice markedly attenuated adipose tissue inflammation as evidenced by reduced tissue lymphocyte infiltration, cytokine expression and NLRP3 inflammasome activity, and improved glucose homeostasis [184]. Furthermore, propionate reduced the proinflammatory response and cytokine secretion induced by LPS in human adipose tissue explants and macrophages [185], whereas butyrate activates the anti-inflammatory Treg cells suppressing the secretion of cytokines via activation of membrane G protein-coupled receptors GPR43/FFAR2 [186]. These receptors, which are mainly expressed in adipose tissue (adipocytes), intestines, and immune cells and signals through G_q_ and G_i/o_, ERK, MAPK, and intracellular Ca^2+^, are important mediators of short-chain fatty acid actions [187]. Noteworthily, as recently reviewed [188], inconsistent results have been obtained in studies with GPR43 deletion in mice, which precludes a full appreciation of the role of these receptors in chronic inflammatory conditions. Finally, short-chain fatty acids were also shown to impair LPS-induced production of nitric oxide and proinflammatory cytokines in the macrophage cell line RAW264.7; such effect seems to involve inhibition of NF*κ*B signaling [189].

In addition to inflammation, short-chain fatty acids also have important effects on energy homeostasis. Recent studies have found that diet enrichment with 5% short-chain fatty acids reduced body weight, adiposity, and hepatic steatosis in diet-induced obese mice by downregulating the activity of the nuclear receptor PPAR*γ* [182], whereas nanoparticle-delivered acetate reduced diet-induced body weight gain, hepatic steatosis, and adiposity by enhancing hepatic mitochondrial function and inducing adipose tissue browning [190].

### 1.11. Newly Identified Lipids and Inflammation

The development of highly-sensitive OMICS techniques (lipidomic, metabolomic, genomic, transcriptomic, epigenomic, and proteomic) in the last decades has allowed the large-scale study of different classes of molecules in distinct compartments and conditions. Among those, metabolomic profiling and lipidomic profiling have emerged as promising methodologies to uncover new metabolites/lipids that either predict or are involved in the development of insulin resistance/T2D. Recently, a metabolomic study of the plasma of 399 nondiabetic subjects with a wide range in degree of insulin sensitivity and glucose tolerance identified a group of three metabolites (*α*-hydroxybutyrate, oleate, and L-glycerylphosphorylcholine) that together could predict the development of insulin resistance [191]. Furthermore, lipidomic characterization of obese, but insulin-sensitive mice, identified a class of endogenously synthesized lipids denominated as branched fatty acid esters of hydroxy fatty acids (FAHFAs) that improves glucose homeostasis and insulin sensitivity and reduces adipose tissue inflammation in diet-induced obese mice [192]. These are only two among many examples of OMICS application to the study of different aspects of obesity-associated inflammation and insulin resistance. These highly sensitive techniques will help us both to unveil the many molecules involved in the development of these diseases and provide major insights in the novel strategies to prevent and treat them.

## 2. Conclusions

There is an established body of literature indicating that nutrients and nutrient sensors are important players involved in the development and maintenance of obesity-associated inflammation and insulin resistance. In these studies, important advances have been made in the characterization of the mechanisms by which nutrients exert their actions, resulting in the unveiling of new therapeutic targets for the treatment of metabolic diseases. Despite this progress, we are far from having the complete understanding about the complex interaction between nutrients and obesity. Indeed, with the advances of techniques such as metabolomics and lipidomics, new nutrient-related molecules are being discovered and characterized for their potential implication in the development of obesity and insulin resistance. The characterization of nutrients and derived metabolites that present proresolution properties towards obesity-associated chronic low-grade systemic inflammation is of major importance if we are to develop novel, effective therapies to prevent metabolic and cardiovascular complications associated with this disease.
